# Vertebral osteomyelitis and native valve endocarditis due to *Staphylococcus simulans*: a case report

**DOI:** 10.1186/1752-1947-2-183

**Published:** 2008-05-29

**Authors:** Natalia Vallianou, Angelos Evangelopoulos, Polyxeni Makri, Georgios Zacharias, Panagiota Stefanitsi, Athanasios Karachalios, Peter C Avgerinos

**Affiliations:** 1 Department of Internal Medicine, Polykliniki General Hospital, Athens, Greece

## Abstract

**Background:**

*Staphylococcus simulans *is a common animal pathogen that occasionally can colonize human skin. Unlike other coagulase-negative staphylococci, *S. simulans *tends to cause more severe infections that resemble those caused by *S. aureus*. We present a case of vertebral osteomyelitis and endocarditis due to *S. simulans*. To the best of our knowledge, this is the first report of vertebral osteomyelitis associated with native valve endocarditis rather than orthopedic surgery.

**Case presentation:**

A 46-year-old male butcher was admitted to the hospital with a 4-week history of high fever with profound sweating. He reported weakness in his legs and low back pain that compromised his walking ability. Blood cultures yielded Gram-positive cocci on Gram stain. These cocci were identified to the species level as *S. simulans*, a coagulase-negative staphylococcus. The patient was treated with antibiotics, which were discontinued after 6 months.

**Conclusion:**

This case illustrates the importance of identifying coagulase-negative staphylococci to the species level. Accurate identification of *S. simulans *would further help investigations defining its pathogenic role in human infections.

## Introduction

*Staphylococcus simulans *is a coagulase-negative staphylococcus, occasionally found on human skin [1]. It is usually acquired from cattle, sheep and their products [1-3]. We describe a case of vertebral osteomyelitis and native valve endocarditis caused by *S. simulans*. To the best of our knowledge, this is the first report of vertebral osteomyelitis unrelated to surgery or orthopedic implant infection caused by this unusual coagulase-negative staphylococcus.

## Case presentation

A 46-year-old male butcher was admitted to the hospital with a 4-week history of high fever with profound sweating. He reported weakness in his legs and low back pain that compromised his walking ability. His past medical history was notable for excessive alcohol consumption, evidence of portal hypertension (ascites, enlarged spleen) and depression. On physical examination, he was fully oriented but had difficulty sitting in the upright position. There was weakness in his legs, whereas his hands had normal strength. No other neurologic deficit was noted. His temperature was 39.2°C and poorly localized tenderness was found over the lumbar vertebrae.

Routine hematological testing showed anemia (hemoglobin 8.5 g/dl), mild thrombocytopenia (platelet count of 132 × 10^3^/mm^3^) and a normal leukocyte count (white blood cell count 8.38 × 10^3^/mm^3^). C-reactive protein (CRP) was 10.50 mg/dl and the erythrocyte sedimentation rate (ESR) was 135 mm/hour. Abnormal liver function tests (international normalized ratio 1.48 and γ-glutamyl-transpeptidase 1050 U/liter), anemia and thrombocytopenia were attributed to excessive alcohol consumption and alcoholic liver disease.

All three sets of blood cultures yielded Gram-positive cocci on Gram stain. Initial identification of these cocci was based on colony and microscopic morphology and a negative coagulase test. These cocci were subsequently identified to the species level as *S. simulans*, a coagulase-negative staphylococcus, by using the Vitek System (bioMérieux S.A., Marcy-l'Etoile, France) [1]. Otherwise, we could have used the ID 32 Staph gallery (bioMérieux) as both systems are based on a series of biochemical reactions and, therefore, have a very good specificity [2]. Minimal inhibitory concentrations (MICs) of the isolate were determined by the broth microdilution method and the results were interpreted according to the National Centre for Surveillance and Intervention (NCSI) recommendations [3]. The MIC for oxacillin was 4 mg/liter.

**Figure 1:**
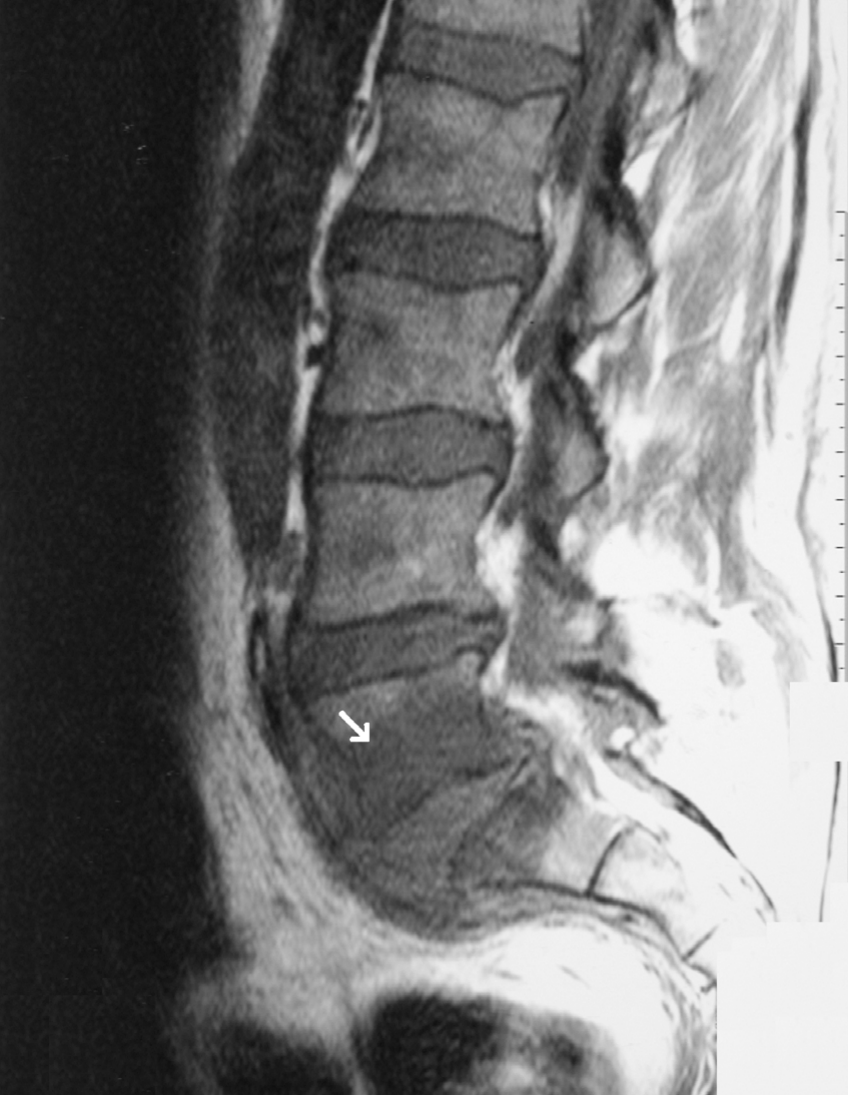
A magnetic resonance imaging scan of the lumbar spine demonstrating abnormal signals of the L5-S1 intervertebral disk. Spinal magnetic resonance imaging revealed diskitis of the L5-S1 intervertebral disk with diffuse edema of the adjacent vertebrae and a paraspinal inflammatory mass with incipient abscess formation.

Computed tomography (CT) and magnetic resonance imaging (MRI) scans of the lumbar spine revealed diskitis of the intervertebral disk at L5-S1 with diffuse edema of the adjacent vertebrae and paraspinal inflammatory tissue with incipient abscess formation (Figure 1). A transthoracic echocardiogram was negative for vegetations, but a transesophageal echocardiogram revealed vegetations of the mitral valve. A CT scan of the brain was performed owing to the patient's gait instability. This showed two hypodense lesions: one in the right parieto-occipital region and one in the right lateral ventricle. These findings were compatible with recent infarcts and were interpreted as septic emboli due to endocarditis.

The patient received a 4-week regimen of vancomycin 1 g intravenously twice a day followed by teicoplanin 400 mg intravenously daily for a total of 5 months. Vancomycin was chosen because the isolated strain was resistant to methicillin. The decision to switch from vancomycin to teicoplanin was because both drugs have a good penetration to bone tissue and teicoplanin can be given once daily, so its use was more convenient. Clindamycin hydrochloride in a dose of 600 mg, orally, three times a day, was added to this regimen after the third month of treatment due to slow progress. The decision to follow conservative treatment rather than surgery was taken because the patient remained afebrile and there was marked improvement of his symptoms. Antibiotics were discontinued after a 6-month course. At that time, the CRP level had returned to normal, the ESR had declined from 135 to 50 mm/hour and there was resolution on the MRI appearances of osteomyelitis. Two months later, the ESR was further reduced and returned to normal.

## Discussion

*S. simulans *belongs to the coagulase-negative staphylococci and is rarely found on human skin [4]. It is a common animal pathogen and is usually acquired from cattle, sheep and other domestic animals [4-6]. Anecdotal reports have associated *S. simulans *with bacteremia, native valve endocarditis, post-surgical pubic osteomyelitis, prosthetic joint infection and urinary tract infection [7-11]. The scarcity of reported human infections caused by *S. simulans *is probably due to the infrequent colonization of human skin by this microorganism as well as the failure of many microbiology laboratories to routinely identify coagulase-negative staphylococci to the species level.

Unlike other coagulase-negative staphylococci, *S. simulans *together with *S. lugdunensis *are more virulent and tend to cause infections that resemble those caused by *S. aureus *[12,13]. Indeed, our patient presented with high fever and native valve endocarditis. There was no history of mitral valve predisposition to endocarditis (for example, mitral valve prolapse). This case is supportive of the observation that infections due to *S. simulans *are reminiscent of those caused by *S. aureus *rather than those caused by most coagulase-negative staphylococci.

The portal of entry of *S. simulans *in this patient remains speculative. Given his profession (butcher), colonization by *S. simulans *may have taken place while working with cattle or sheep. Suppressed immunity, owing to impaired liver function, may have contributed to the severity of his infection. Therefore, our patient's profession and history of alcohol consumption provide some clues to the means of acquisition and spread of *S. simulans*. To the best of our knowledge, this is the first report of vertebral osteomyelitis associated with native valve endocarditis rather than orthopedic surgery.

## Conclusion

This case illustrates the importance of identifying coagulase-negative staphylococci to the species level. Accurate identification of *S. simulans *would help further in defining its pathogenic role in human infections.

## Abbreviations

CT: computed tomography; CRP: C-reactive protein; ESR: erythrocyte sedimentation rate; MIC: minimal inhibitory concentrations; MRI: magnetic resonance imaging.

## Competing interests

The authors declare that they have no competing interests.

## Consent

Written informed consent was obtained from the patient for publication of this case report and any accompanying images. A copy of the written consent is available for review by the Editor-in-Chief of this journal.

## Authors' contributions

NV, PM, GZ, PS, AK and PCA made substantial contributions to the conception of the study, and analysis and interpretation of data, AE was involved in reviewing the laboratory data, drafting and revising the manuscript. All authors gave final approval of the version to be published.
